# Understanding the biology of mucormycosis using contemporary omics tools: a review

**DOI:** 10.5114/bta/220617

**Published:** 2026-06-12

**Authors:** Dhananjaya Mishra, Smaranika Pattnaik

**Affiliations:** 1 Department of Biotechnology and Bioinformatics, Sambalpur University, Burla, Odisha, India; 2 Laboratory of Medical Microbiology, School of Life Sciences, Sambalpur University, Burla, Odisha, India

**Keywords:** mucormycosis, omics, virulent genes

## Abstract

Mucormycosis (the “black fungus infection”) is a life-threatening angioinvasive fungal infection caused by opportunistic fungi of the class *Mucormycetes*.

A lot of clinical exercises were made in the post-coronavirus disease 2019 period due to admission of patients suffering from so called “black fungus disease”. Many of patients had been operated for excision of their vital organs, colonized with massive fungal mycelia to restrict further invasion. In these circumstances, this apt article endeavour to scrutinize and debate some of the viable factors and prospective mechanisms that can assist to understand and elucidate the enigma of sudden, steep and deadly upsurge of mucormycosis infection. Here we review the specific contribution of high-throughput next-generation sequencing and multi-omics-based approaches to the general knowledge and understanding of *Mucormycetes* and further detail about the most exciting discoveries pertaining to the recently identified genetic advancements in *Mucormycetes* and mucormycosis. Through the use of a few genetic study models virulence factors in *Mucormycetes* that were previously unknown have been identified. Most remarkably, new research has opened up new possibilities for developing novel treatments against mucormycosis by identifying novel genes and process governing the pathogenic potential of *Mucormycetes* and their interaction with host. Ultimately virulence investigations in *Mucormycetes* that were previously hindered are now possible with new study models indicating a promising future for the field leading to the development of therapies to treat mucormycosis.

## Introduction

Fungal infections have emerged as significant global challenges in recent decades, as a wide range of fungal co-infections have been identified in conjunction with medical emergencies such as pandemics, endemics, and epidemics. In clinical settings, it has been observed that various infections caused by bacterial or viral pathogens tend to progress into pandemic scenarios (Seyedjavadi et al. 2022). Moreover, it has been frequently reported that the onset of such diseases can lead to the emergence of co-infections or post-infections, typically caused by opportunistic fungal pathogens (Al Balushi et al. 2022). These opportunistic fungal agents can effectively penetrate host tissues by evading the actions of immune cells mobilized by the human immune system (Kobayashi 1996). Consequently, it is essential to restrict fungal invasion following illness-related infections through single or multi-metric approaches.

Approximately 6.5 million individuals are affected by invasive fungal infections annually, and about 3.8 million individuals die due to infection-related complications. Of these, approximately 2.5 million deaths can be directly attributed to fungal infections (Denning 2024). Recent studies have shown that the epidemiology of fungal infection is constantly evolving and is influenced by new emerging hazards associated with the coronavirus disease 2019 (COVID-19) pandemic, transfer of pathogens, and newly developed resistance to antifungal agents (Lass-Flörl and Steixner 2023).

Angioinvasive fungal infections (AFIs) pose a significant threat due to their propensity to infect blood vessels, leading to severe consequences of destructive tissue ischemia, infarction, and necrosis (Shields et al. 2019). Following fungal infection in the bloodstream, the resulting pathological changes can lead to multi-organ failure (Mihailides et al. 2020), ultimately leading to death. The fungi most commonly implicated in AFI include *Aspergillus*, *Candida*, *Fusarium*, *Mucormycetes*, and various molds (Gaillard and Jones 2010).

The occurrence of viral infections in immunocompromised individuals markedly elevates the immune response. This elevated response involves the activation and sensitization of immune cells such as macrophages and dendritic cells, along with the induction of cytokine release and the overexpression of virulence factors. These responses create favorable conditions for immune suppression. Because of their opportunistic nature, fungi utilize these conditions to penetrate host tissues, evade immune responses, and initiate intracellular spore germination or hyphal elongation, thereby producing toxic substances to enhance their pathogenicity (Dropulic and Lederman 2016; Marcos et al. 2016; Sephton-Clark and Voelz 2017; Li et al. 2019; Lionakis et al. 2023; Stuckey and Santiago-Tirado 2023; Anandani et al. 2025).

Building further on this context, while fungal infections may range from superficial to invasive, certain opportunistic fungi such as *Mucorales* can pose significant threats to immunocompromised patients. This group of infections includes mucormycosis, a rare but aggressive fungal disease that primarily affects individuals with uncontrolled diabetes, prolonged corticosteroid exposure, or weakened immunity, leading to rapid tissue death and elevated morbidity (Ghuman and Voelz 2017; Steinbrink and Miceli 2021; Monika and Chandraprabha 2022).

## Mucormycosis

At this point, it is imperative to expand upon the previously mentioned details. Mucormycosis is a progressive and life-threatening AFI caused by various fungal species belonging to the subphylum *Mucormycotina* and the order *Mucormycetes* (Ribes et al. 2000; Spellberg et al. 2005). The members of *Mucormycetes* responsible for causing mucormycosis are often referred to interchangeably as *Mucormycetes* or mucormycosis-causing fungi. The term “black fungus” is commonly used to describe human pathogenic *Mucormycetes* species, based on the black-colored sporangia produced by these fungal species.

*Mucormycetes* are classified as opportunistic pathogens that typically cause infections under immunocompromised conditions. Therefore, they are considered the third most common cause of AFI in immunocompromised populations (Millon et al. 2016). Potentially lethal mucormycosis infections can be initiated through ingestion, inhalation, or contamination of wounds with aerosolized spores from the environment. Consequently, these infections are associated with excessive morbidity and mortality rates, often exceeding 50% and approaching 100% with disseminated infection, despite aggressive tissue debridement and antifungal therapy (Jerez Puebla 2012; Katragkou et al. 2014).

*Mucormycetes* are rare yet opportunistic pathogens that mainly affect immunocompromised individuals. Their tendency for aggressive invasion of blood vessels and vascular tropism leads to tissue infarction. People with immune system-associated comorbidities, such as diabetes, neutropenia, iron overload, deferoxamine therapy, renal failure, protein-calorie malnutrition, and other diseases, are particularly highly susceptible to develop mucormycosis.

Currently, 27 *Mucormycetes* species across 11 genera have been identified as causative agents of mucormycosis. *Rhizopus* species are the most common causative agents, accounting for approximately 70% of all cases, and they are the most common organism isolated from mucormycosis patients (Soare et al. 2020). In India, *Rhizopus arrhizus* is the most common species causing mucormycosis in humans, followed by *Apophysomyces variabilis*, *Rhizopus microporous*, and *Rhizopus homothallicus* (Divakar et al. 2021).

### Genes linked to pathogenic Mucormycetes

The pathogenic strains of *Mucormycetes* possess specific genes that are expressed before the onset of fungal pathogenicity. As reported earlier, the spore-coat protein homolog (*cotH*) gene is expressed during intracellular invasion (Lackner et al. 2021; Cánovas-Márquez et al. 2023). The cotH3 and cotH4 proteins are specifically associated with virulence under conditions of diabetic ketoacidosis, as demonstrated in murine models.

Mucormycosis-causing fungi are known for their ability to sequestrate iron in intracellular environments. In these species, iron uptake is mediated by *ftr1* and *fet3* genes. The former functions as “iron permease,” while the latter encodes the ferroxidase enzyme (Lax et al. 2020). Additionally, the gene encoding calcineurin has an active role in the pathogenesis of dimorphic *Mucormycetes*; this is because the calcineurin signaling pathway plays a critical role in regulating hyphal morphology required for fungal colonization (Lee et al. 2013; Homa et al. 2022). During stress conditions, the fungal cell expresses calcineurin, which enables the cell to attain a yeast cell-like morphology that persists during unfavorable conditions. Calcineurin is produced following stress mitigation. Another gene namely histone methyltransferase Set1, is involved in epigenetic changes in DNA and induces DNA methylation; mutational studies have shown that this gene mediates inhibition of host DNA repair (Osorio-Concepción et al. 2023). Furthermore, non-canonical RNAi Pathway Components, r3b2 (ribonuclease III-like), and rdrp1 (RNA-dependent RNA polymerase) can control gene expression through an epigenetic regulatory mechanism, demonstrating resistance toward conventional antifungal agents (PerezArques et al., 2020)

## Resistance of *Mucormycetes* to conventional antifungal agents

*In vitro* studies (Borman et al. 2021) have revealed that *Mucormycetes* exhibit resistance to conventional antifungal agents (particularly newer triazoles). In a review report, Dannaoui (2017) mentioned homogeneity of mucormycoses and cumulative drug resistance conferred by different strains. However, the onset of COVID-19 pandemic further triggered the topic of drug resistance in clinical settings, as mucormycosis was recorded as the secondary infection during the post-therapy period.

## COVID-19-associated mucormycosis and antifungal resistance in *Mucormycetes*

Following its emergence in late 2019, the severe acute respiratory syndrome coronavirus 2 (SARS-CoV-2) virus has severely disrupted the global healthcare system and inflicted massive damage on the world economy. COVID-19 has affected over 220 countries and territories, resulting in approximately 7,010,681 deaths worldwide as of March 2025 (Worldometers 2025). The waves of COVID-19 infection, along with the emerging variants of SARS-CoV-2, have not only impacted global public health, but they have also triggered a dramatic increase in fatal fungal infections in infected individuals, thereby posing a life-threatening risk (Revannavar et al. 2021; Werthman-Ehrenreich 2021).

Post-COVID-19 immune dysregulation has significantly increased the susceptibility of individuals to opportunistic infections, with mucormycosis emerging as a major concern. The widespread use of corticosteroids, particularly during COVID-19 treatment, combined with pre-existing conditions such as diabetes, creates a hyperglycemic and immunocompromised environment that is highly conducive to fungal invasion. *Mucorales* species exploit these vulnerabilities, often causing rapid tissue necrosis and severe morbidity as mentioned earlier. The alarming increase in post-COVID mucormycosis cases has highlighted the critical need for early diagnosis, personalized antifungal therapy, and strict glycemic control to reduce the impact of this disease.

Azole-class antifungal agents are the most commonly used medications for treating fungal infections. Among these antifungal agents, triazoles are compounds characterized by a cyclic structure containing three nitrogen atoms. They inhibit the activity of cytochrome P450 enzymes, which play a crucial role in synthesizing ergosterol, a vital component of fungal cell membrane. Studies have shown that antifungal resistance in *Aspergillus* species is largely mediated by mutations or overexpression of the cytochrome P450 enzyme Erg11 (Caramalho et al. 2017).

A potential explanation for the aforementioned coinfections is the dysregulation of cytokine and interleukin production. The synthesis of various cytokines is significantly upregulated, culminating in an event termed “cytokine storm” (Chakrabarti 2021). Other researchers have suggested that a delayed type-I interferon (IFN) response due to COVID-19 infection creates a favorable environment for fungal invasion.

Brodin (2021) explained that viral infection alters cellular responses, inducing increased reactive oxygen species (ROS) production, calcium flux, protein aggregation, and notably, activation of the NLRP3 (NLR family pyrin domain-containing 3) inflammasome. This activation leads to caspase-1-dependent cleavage and release of proinflammatory cytokines (interleukin-1β and interleukin-18). Consequently, Th1 immune cells and NK cells secrete IFN-γ, further delaying the type-I IFN response. These immunological events create an opportunity for fungal hyphae to penetrate host tissues. This situation is further complicated by the depletion of cluster of differentiation 4- and 8-positive T cells, which significantly weakens immune defense. This reduction in the cellular immunity level allows opportunistic *Mucormycetes* to invade target cells, particularly in the lungs and alveolar spaces, which are the most common sites of mucormycosis infection (Bhatt et al. 2021; Sarkar et al. 2021).

Additional fungal co-infections were observed following SARS-CoV-2 infection. Cryptococcosis was reported in some cases (Walker et al. 2024), while co-infection with *Candida* species in COVID-19 patients with severe pneumonia was also documented (Segrelles-Calvo et al. 2021). However, determining the true prevalence of mucormycosis remains difficult. This is largely due to the lack of mandatory reporting, absence of national surveillance systems, difficulties in achieving diagnostic accuracy, and a steady decline in autopsy rates. Together, these factors likely contribute to an underestimation of the actual burden of mucormycosis (Walsh et al. 2014).

## Morbidity and mortality

Based on the preceding points that highlight the aspects of morbidity and mortality due to mucormycosis, it is noteworthy to further emphasize the overall sense of severity of impact. A considerably higher incidence of mucormycosis has been reported in India compared to that in other countries. The estimated prevalence of mucormycosis in India is approximately 140 cases per million population, which is roughly 80-fold higher than that in developed countries such as the United States and Europe. The mortality rate is also correspondingly high. A case-control study conducted at a multi-specialty tertiary care facility in western India documented 73 patients with post-COVID-19 rhino-orbit-cerebral mucormycosis (ROCM), of which 36% patients died due to the disease within 10 days of admission. The cumulative mortality rate had increased to 53% by day 21 (Choksi et al. 2022). A thorough literature review by Prakash and Chakrabarti (2021) emphasized the heterogeneity of the death rates according to the form of mucormycosis: 31–49% due to ROCM, 61–77% due to the pulmonary form, 23–57% due to cutaneous infection, 67–94% due to the gastrointestinal form, and 62–79% due to disseminated infection. In 2019, Nucci et al. (2019) conducted a literature surveillance study of mucormycosis cases published between 1960 and 2018; they found that 6 and 51 mucormycosis cases were reported in the United States during the 1960s and the 2010s, respectively. In another study of 143 South American cases, 20% cases were associated with skin-penetrating trauma/burns, while 42% cases had diabetes mellitus as a comorbidity; additionally, 45.5% patients had immunosuppressive conditions such as corticosteroid therapy, solid organ transplant, and therapy for hematological cancer (Nucci et al. 2019).

According to a World Health Organization survey (2025), disseminated mucormycosis had the highest fatality rate of up to 68%, as the infection can spread to more than one organ. Compared to India and South America, Europe has reported a lower incidence of mucormycosis. Furthermore, based on research conducted by the Centre for Disease Control and Prevention (2024), mucormycosis accounted for only 2% of all invasive fungal infections among recipients of solid organ transplant in the United States. The most common underlying causes of mucormycosis among the European population are solid organ transplants and hematologic malignancies, where the highest death rates were reported as the infection had disseminated to multiple sites.

Because mucormycosis associated with COVID-19 is a serious condition, numerous targeted therapeutic developments are being investigated to mitigate the severity of such opportunistic infections. Recent developments involve enhanced drug formulations for antifungal treatment, application of immunomodulatory therapy, and advanced surgical techniques, all of which have proved to be promising to reduce the disease burden. Additionally, new technologies such as genomics, transcriptomics, and proteomics are currently transforming the landscape of mucormycosis diagnosis and management. With the integration of molecular information and clinical strategies, clinicians can attain increased diagnostic accuracy and obtain higher-quality treatment results. These developments are an encouraging step forward toward enhancing the survival rate and overall care for mucormycosis patients. Hence, potential therapeutic strategies are recommended.

## Potential therapeutic strategies

An important strategy for treating mucormycosis is the early and precise diagnosis, preferably through assays of the body fluid or serum to identify certain biomarkers. Following diagnosis, the disease course can be controlled using prompt curative or preventive treatments. At an advanced disease stage, the typical approach is surgery along with specific antifungal drugs (Alqarihi et al. 2023). Because corticosteroids are commonly administered to individuals with COVID-19, their immunological parameters should be continuously monitored. Corticosteroid use has been reported to cause immunosuppression – a predisposing factor for fungal invasion. Therefore, it might become essential to strengthen the immunity, either through active or passive immunocyte therapy. Maintaining a normal blood glucose level is also equally important (Aranjani et al. 2021). These measures are part of the overall “management of comorbidities.” Another promising area is the development of new drug delivery systems, particularly nanoparticle-based antifungal therapies with greater therapeutic indices.

However, despite these advancements, further investigations are required to reveal the molecular mechanisms underlying the pathogenesis of *Mucormycetes*. Compared to prominent pathogens such as *Candida albicans* and *Aspergillus fumigatus*, there has been limited research on the genetic makeup of *Mucormycetes*. *Mucormycete* fungi are estimated to have diverged from fungi such as *Ascomycetes* and *Basidiomycetes* over 800 million years ago; consequently, this long evolution period prohibits the establishment of parallels and the transfer of insights from model organisms to the study of mucormycosis (Garcia et al. 2018).

## Comprehending the biology of *Mucormycetes* through omics techniques and tools

**Figure 1:**
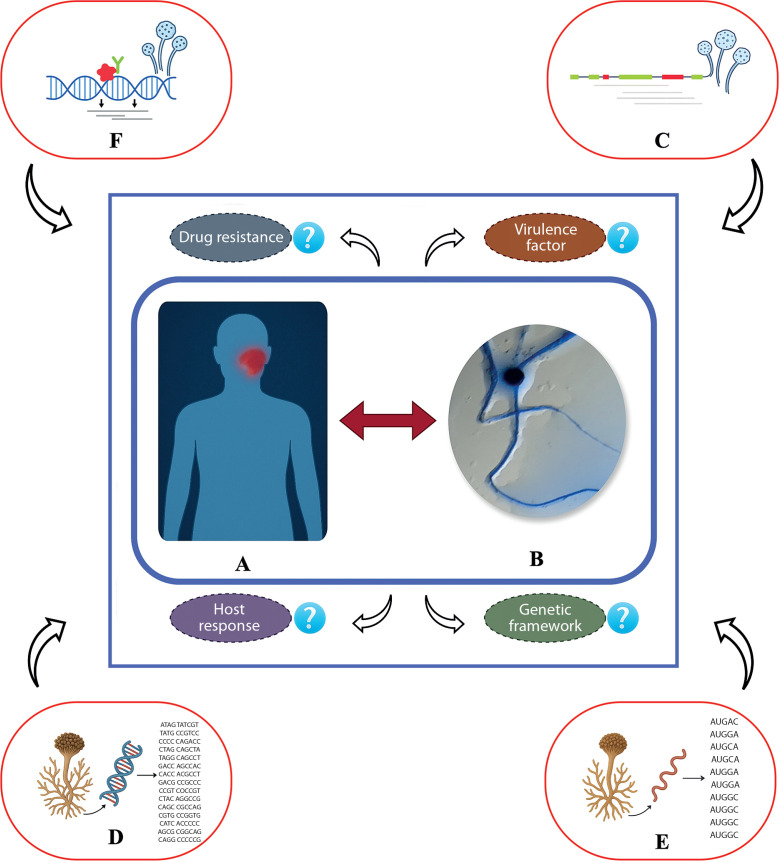
The host–pathogen interaction. (**A**) Symptomatic host. (**B**) Invading fungal mycelia with black spores. (**C**) 18S ribosomal RNA signature sequencing. (**D**) Whole genome sequencing. (**E**) Transcriptomics of messenger RNA. (**F**) Chip-sequencing technique involving nucleic acid-protein interaction with cognate antibodies. Contemporary omics-based strategies, including genomics, transcriptomics, proteomics, and metabolomics, are increasingly being employed for both prognosis and diagnosis of mucormycosis, as they provide comprehensive insights into host-pathogen interactions, which play a pivotal role in disease onset and progression

The study of pathogenic mechanisms of *Mucormycetes* is complicated by various factors such as their genetic intractability, evolutionary distance, and the restricted use of available genetic tools. These limitations are responsible for the current gap in knowledge. Therefore, these challenges need to be overcome to better understand mucormycosis and develop potent diagnostic and therapeutic measures. The deficiencies of classical forward and reverse genetics can be mitigated using unbiased high-throughput next-generation sequencing (NGS) and omics techniques to reveal the pathogenic mechanisms of *Mucormycetes* (Figure 1).

The Figure depicts the overview of omic-based approaches to detect acute and chronic mucormycosis. The initial step is 18S ribosomal RNA (rRNA) sequencing of causative fungi. This conserved RNA sequence is amplified by polymerase chain reaction (PCR), followed by sequencing of the PCR product through Sanger’s sequencing technique. The obtained sequence is then subjected to the Basic Local Alignment Search Tool (nucleotide) (BLASTn) program at the National Center for Biotechnology Information (NCBI) website (https://www.ncbi.nlm.nih.gov/) to examine absolute similarity. A phylogram is created to interpret the genetic relationship with the related fungi, complementing information for the diagnosis of mucormycosis in clinical practice (Hammond et al. 2011; Alanio et al. 2015; Safiia et al. 2024).

Whole genome sequencing (WGS) through NGS technologies delivers high-quality sequence information at the codon level, facilitating gene identification and understanding of drug resistance mechanisms. WGS information can be screened for gene identification by using programs such as ORF Finder. Open reading frames are nucleotide sequences that do not contain stop codons between the start and terminal codons. Additionally, by utilizing the Conserved Domain search tool on the NCBI website, a more comprehensive analysis can be performed to recognize conserved protein structures, categorize proteins into families, and obtain information regarding their functions and potential structural characteristics. These analyses provide insights into the structural and functional characteristics of query proteins (Shelburne et al. 2015; Garcia-Hermoso et al. 2018; Michael et al. 2023; Conlan et al. 2024).

WGS has gained popularity as an effective and robust tool in research on *Mucormycetes* to facilitate accurate species identification, support epidemiological research, and define genetic determinants of virulence and antifungal susceptibility. The source-tracking of nosocomial or environmental clusters can be performed by resolving strains and species-level relationships that cannot be deciphered by Internal Transcribed Spacer or single-gene markers (e.g., genomic analyses of *Rhizopus* isolates of COVID-era Rhino-cerebral mucormycosis) (Michael et al. 2023).

More recently, long-read and hybrid assemblies have provided more high-quality reference genomes of medically relevant *Mucor* species, including detailed gene catalogues. This has enhanced the annotation of mitochondrial elements, secondary metabolite clusters, and putative virulence factors (Buddie et al. 2025; Van Den Ende et al. 2025).

WGS integrated with metagenomic NGS (mNGS) in clinical microbiology supplements traditional culturebased and targeted PCR diagnostics by enabling the detection of mixed or cryptic infections and providing high-resolution epidemiological data. However, WGS/mNGS technology has not been widely applied in clinical practice due to various obstacles, including prolonged turnaround time, requirement for a well-developed bioinformatics framework, and difficulty in interpreting the results (Safiia et al. 2024; Zhang et al. 2024).

Despite the promising potential of genomic methodologies, there has been limited systematic research on *Mucorales* due to various factors. The genomes of *Mucorales* are often large, highly repetitive, and not well represented in existing reference repositories, making them challenging to assemble genomically, predict genes accurately, and identify resistance markers. Moreover, high sequencing cost, risk of inherent contamination, and absence of standardized systems and frameworks to support clinical validation remain barriers to the extensive clinical adoption of these genomic methods (Gullí et al. 2024; Tomer et al. 2024).

Taken together, recent research has confirmed that WGS is reinventing the biology of *Mucormycetes* and the mechanism of mucormycosis outbreaks. The broad use of this technology for diagnosis and surveillance, however, requires the curation of wider reference collections, standardized analytics pipelines, and accelerated workflows that need clinical confirmation (Chibucos et al. 2016).

RNA sequencing (RNA-seq) provides the transcriptional output of the genome and thus reveals important gene expression profiles. The reverse transcription of RNA into complementary DNA allows precise identification and functional characterization of genes, which facilitates the deciphering of the regulatory mechanisms and cellular pathways. Transcriptomics techniques can provide definitive evidence of mucormycosis (Chibucos et al. 2016; Lebreton et al. 2019; Soare et al. 2020; Alqarihi et al. 2023; Zhang et al. 2023).

Here, a notable aspect is that both innate and adaptive immune factors contribute to the severity of mucormycosis. B and T cell function should be closely monitored, and immunoassays supported by omics strategies such as antigen–antibody interactomics should be used. It is equally important to consider patient history. Furthermore, AI-assisted banks can help clinicians with diagnosis (Cánovas-Márquez et al. 2023; Safiia et al. 2024; Brown et al. 2025).

Extracellular RNA (eRNA) and interfering RNA (RNAi) are useful for disease detection and regulation. eRNAs, isolated from bodily fluids, can harbor mucor fungal genes and can be sequenced for disease diagnosis. RNAi enables to conduct gene knockout of virulence genes through the use of omics technologies (Chettimada et al. 2020; Perez-Arques et al. 2020; Soare et al. 2020).

Recent studies using RNA-seq to analyze mucormycosis have better understanding of the transcriptional responses of the host and pathogen, although such studies remain limited compared to research targeting other fungal pathogens. Notably, whole-genomic and transcriptomic characterization using dual-species RNA-seq of *Rhizopus delemar* and *Rhizopus oryzae* during their interactions with human airway epithelial cells revealed sets of fungal genes that were upregulated during the early phases of infection (6 and 16 h), suggesting their potential roles during virulence (Chibucos et al. 2016).

Regarding host response, transcriptomic profiling revealed mechanisms of immune dysregulation in COVID-19-related pulmonary mucormycosis (CAPM). A study analyzed RNA-seq data of monocytes and neutrophils of patients with and without COVID-19 and found that CAPM was characterized by the dysregulated expression of pattern recognition receptors such as dectin-2 and C-type lectin family proteins; enhanced NETosis; metabolic reprogramming, including glycolysis, pentose phosphate pathway, and iron metabolism/ferroptosis; and abnormal induction of proinflammatory mediators (Dhaliwal et al. 2023).

Another pertinent study investigating comparative host transcriptome in response to pathogenic fungi revealed common and species-specific transcriptional antifungal host response pathways compared to monocyte responses with peripheral blood mononuclear cells following exposure to *R. oryzae* and other opportunistic pathogenic fungi. The authors showed that after induction with *R. oryzae*, the IFN type I and II signaling pathways were mildly activated; however, strong changes were noted in glycolysis activity, accumulation of ROS, and markers of endoplasmic reticulum (ER) stress response (Bruno et al. 2020).

ChIP-sequencing (ChIP-seq) is used to explore the interaction of proteins with DNA, including histone modifications and binding of transcription factors. This method identifies epigenomic modifications and regulators of pathological gene expression (Lupfer et al. 2014; Wang et al. 2024).

A critical issue is the emergence of novel virulent genes (NVGs) with a crucial role in the pathogenesis of mucormycosis. Therefore, bioinformatic tools should be used to detect NVGs.

For *Mucormycetes* such as *Mucor*, *Rhizopus*, and *Lichtheimia*, ChIP-seq can reveal the mode of regulation of virulence traits (such as spore germination, angioinvasion, or immune evasion) by transcription factors, regulation of chromatin modifications by environmental stress factors (such as hypoxia, oxidative stress, or iron deficiency), and the mode of epigenetic modifications to induce drug resistance or pathogenicity (Navarro-Mendoza et al. 2019; Osorio-Concepción et al. 2024; Zhang and Tao 2025).

To date, few studies have applied ChIP-seq directly to *Mucormycetes*. Model or opportunistic fungal species such as *Aspergillus*, *Neurospora*, *Candida*, and *Zymoseptoria* have been utilized in the majority of studies on fungal ChIP-seq. ChIP-seq with antibodies against histone markers (H3K4me2 for euchromatin and H3K9me3 and H3K27me3 for heterochromatin) has been applied to map chromatin across *Zymoseptoria tritici* strains. These are informative methodological examples that can potentially be applied to adapt protocols to *Mucormycetes* (Soyer et al. 2015).

In another study, centromeres of *Mucor circinelloides* were mapped by ChIP-seq, and a mosaic-like organization of the centromere was reported. More recently, ChIP-seq was used to profile H3K9me3 and its correlation with adenine methylation in *Rhizopus microspores*; the results enabled to identify developmental silencing of genes (Navarro-Mendoza et al. 2019; Lax et al. 2025). Thus, these studies illustrate how ChIP-seq can be used to define epigenetic landscapes and downstream transcriptional regulators in early-branching fungi by using similar methodological templates developed for other filamentous fungus (Furey 2012; Chung et al. 2014).

However, the application of ChIP-seq for research on *Mucormycetes* has several challenges and limitations. A major barrier is the availability of optimal antibodies against transcription factors or histone modifications from these organisms. Moreover, immunoprecipitation is non-feasible (or non-stoic) because there are no epitope-tagged strains or antibodies that were tested against macrophages.

Another constraint is that some *Mucormycetes* have genomic content that frequently comprise incomplete or poor-quality assemblies; this impedes performing short read mapping, worsens peak calling issues, and hampers the characterization of regulatory regions. Additionally, *Mucormycetes* may have chromatin or cell wall traits (for example, cell wall rigidity or chromatin variation) that hinder efficient extraction and crosslinking and chromatin fragmentation. Additionally, within infection models (tissues and host interactions), unknown or very low amounts of fungal biomass can reduce background signals and result in noisy or shallow data. Lastly, several transcriptional or epigenetic alterations depend on specific conditions (for example, time, stress, and host environment); this could accentuate the need to control strenuously, make replications, and design experiments with care (e.g., input DNA and immunoglobulin G controls) (Soyer et al. 2015).

Despite these limitations, based on the increasingly available genomic resources and several published ChIP-seq datasets (NCBI GEO Accession Number: GSE276938), ChIP-seq has provided substantial insights into the mechanism of chromatin organization and regulation in *Mucormycetes*. Therefore, this method has high potential for application in the investigation of pathogenesis and host-pathogen interaction during mucormycosis.

## Detection of NVGs using omics tools and techniques

Most genetic investigations discussed in the preceding sections focused on the function of genes identified in virulent *Mucormycetes*. Recent advancements in omics technologies have facilitated to conduct more ambitious research for discovering novel virulence factors in *Mucormycetes*. Comparative genome studies of an avirulent strain and a virulent strain of *Mucor lusitanicus*, i.e., NRRL363 and CBS277.49, respectively, showed that the avirulent strain lost 543 genes and contained 230 disordered protein-coding genes with genome variations characteristic of their individual pathogenicities (Lopez-Fernandez et al. 2018).

Subsequent functional analysis of these genetic differences identified a secreted protein with an unknown function that was confirmed to play an essential role in the pathogenicity of the CBS277.49 strain. A similar strategy applied at the RNA level yielded a novel exonuclease termed Wex1, which was revealed through the transcriptome analysis of the virulent strain CBS277.49 and the avirulent strain NRRL363. This enzyme was observed to be involved in pathogenicity. Additionally, the use of a high-throughput RNAi library and a functional genomics approach enabled researchers to screen efficiently for new virulence factors. A collection of plasmids that can silence each gene in *M. lusitanicus* allowed to isolate transformants with notable traits. This process identified mcpID and mcmyo5 as novel pathogenic factors in *Mucormycetes* (Trieu et al. 2017).

Researchers also reported on the roles of several other genes, including *gcn4*, a transcription factor; *hmgR*, which encodes 3-hydroxy-3-methylglutaryl coenzyme A reductase; and *igp1*, an immunoglobulin-like protein that interacts with host cell receptors (Zhang et al. 2023).

In *Mucorales*, including *R. delemar* and *M. circinelloides*, the conserved transcription factor Gcn4 is the master regulator of amino acid starvation response and is induced during host-related nutrient deficiency. By promoting biosynthetic and metabolic processes associated with amino acids, Gcn4 enables fungal cells to sustain their biosynthetic processes and growth even during extracellular amino acid shortage, such as within phagosomes or nutritionally stressed host tissues. This Gcn4-mediated transcription program reveals a mechanism through which *Mucormycetes* respond to host-imposed nutrient constraints that comprise part of “nutritional immunity”; moreover, this adaptive mechanism potentially contributes to fungal survival during the infectious process and acts as a potential virulence factor. These interpretations were derived based on corroborative evidence presented by transcriptomic and functional studies that showed Gcn4 induction during phagosome-host interactions and general studies on Gcn4/ATF4 networks that determined its defining role in amino acid biosynthesis induction during starvation (Pérez-Arques et al. 2020).

In *M. circinelloides* and other Mucorales, the *hmgR* gene encodes for 3-hydroxy-3-methylglutaryl-CoA reductase (HMGR), the key enzyme of the mevalonate pathway that transforms HMG-CoA to mevalonate, which is a precursor to sterols and other isoprenoids. This pathway is essential for the synthesis of ergosterol, an important constituent of the fungal cell membrane that maintains membrane stability, fluidity, and permeability and contributes to growth, spore viability, and survival under environmental stress. The loss of *hmgR* function can weaken membrane stability and reduce the survival of fungal cells within host tissues. Moreover, *hmgR* overexpression can contribute to azole resistance through the activity of HMG-CoA reductase to maintain ergosterol synthesis against antifungal pressure. Thus, *hmgR* is a metabolic and drug resistance-associated virulence factor (Nagy et al. 2019).

In *M. circinelloides* and other *Mucorales*, the *igp1* gene encodes for a surface protein that contains an immunoglobulin-like (Ig-like) domain. By interacting with host receptors, Igp1 can potentially enhance fungal colonization, immune evasion, or invasion. The presence of its Ig-like structure suggests potential exploitation of molecular mimicry, enabling the fungus to interact with host cells and potentially make itself undetected by the immune system. Thus, by functioning as a key virulence factor, Igp1 enables adhesion, tissue colonization, and immune evasion, all of which contribute to play a significant role in the pathogenicity of *Mucormycetes*. The structural mimicry of host immunoglobulins helps the fungus to regulate and direct host-fungal interactions to its benefit (Tahiri et al. 2023).

In the given context, the application of high-throughput computational analysis of the entire set of genomes of interest, data mining, and creating public databases are of immense importance.

## Computational tools for the analysis of *Mucormycetes*

Because *Mucormycetes* are filamentous fungi, they have a complex genome architecture containing eukaryotic exon–intron signature sequences. Moreover, the intracellular invasion by the candidate fungus creates severe anomalies in genome biology. The advent of computational tools and techniques together with dataset resources have facilitated genome mining studies, although the absolute performance of these tools remains questionable. These tools include genome assembler – a software for genome assembly, SPAdes – used for assembly of short reads eluted from the fungal genome (Bankevich et al. 2012), Canu – used for assembly of long reads of genome by utilizing the platform of PacBio and Oxford-Nanopore (Koren et al. 2017), and Trinity – a portal for transcriptomics assembly and whole-genome assembly tasks (Grabherr et al. 2011).

The tools for genome annotation can also be availed. AUGUSTUS is a program for the prediction of the gene of interest and for structural and functional annotation strategies (Stanke et al. 2004). Because *Mucormycetes* are eukaryotic fungi, GeneMark can be used as a pertinent tool for gene prediction (Besemer et al. 2005), while fungal genome annotations can be performed using Prokka – a prokaryotic genome annotation server (Seemann 2014). Considering the comparative genomics approach for fungal genome, Mauve is useful for aligning similar genomes and inferring about putative genetic linkages among the studied gene sequences (Darling et al. 2004). BUSCO can also be utilized for assessing genome completeness and for detecting conserved domains (Simao et al. 2015).

Coding sequence search on the NCBI portal can also be used to detect conserved domains and their membership in protein families and protein superfamilies (Furuno et al. 2003). The evolution of fungal genome architecture is an important aspect of fungal biology. Therefore, programs such as RAxML and IQ-TREE-2 were developed for phylogenetic analyses considering the maximum likelihood method for linkage studies (Stamatakis 2014; Minh et al. 2020).

Host factors combined with environmental stress such as antifungal pressure can induce the fungal genome to undergo genome plasticity and acquire resistant genes, a prominent phenomenon in clinical settings. Therefore, an approach termed variant calling was developed. Genome Analysis Toolkit (GATK) and BCF tools are useful for recognizing variations in genomic data and for analyzing vcf (Variant Call Format) files, respectively (Bathke and Lühken 2021; Danecek et al. 2021).

Functional annotation is an important phase in genome science. BLAST tools are generally used for sequence alignment, and the aligned sequences can be functionally annotated against public databases. InterProScan facilitates the identification of protein domains and annotation functionally (Quevillon et al. 2005). Data visualization is a mandatory step for comparative genomic studies. In this context, Integrative Genomics Viewer and Circos are used for whole genome visualization in linear and circular forms, respectively (Krzywinski et al. 2009; Robinson et al. 2011). The Gene 112092 tool can detect an extracellular protein of unknown function from a query sequence (Zhang et al. 2023).

## The omics-based host response system

Host response to mucormycosis is critical to control the degree and severity of infection. Hence, omics tools and techniques are exploited during prognosis and diagnosis of mucormycosis. Transcriptomics-based tools can decipher the signaling pathways [such as Epidermal Growth Factor Receptor (EGFR)-based signaling activation], which is considerably related to fungal invasion and host cell response (Soare et al. 2020). Protein and DNA microarray (using robotics) of host cells has revolutionized the clinical treatment of fungal infections. Additionally, genomics, proteomics, and metabolomics can determine the status of the host response system during mycotic invasion (Gu et al. 2025).

Based on recent findings, it can be inferred that, together with conventional virulent factors such as aflatoxin (Seyedmousavi et al. 2018), several other factors also play a key role in the pathogenesis of mucormycosis. Alqarihi et al. (2023) documented host EGFR overexpression with epigenetics change evidenced by phosphorylation. Enzymes, such as iron reductases (Fre), a ferroxidase (Fet3), and a high-affinity iron permease (Ftr1), are also considered virulent factors because they facilitate the acquisition of iron from host hemoglobin, following infection with *Mucormycetes* members; this phenomenon explains the angio-invasive activity of the invading fungus. “Morphogenic switches” are also considered virulent factors because the invasive fungal strains prefer to assume a filamentous form during host invasion. Therefore, the fungus changes its spore morphology to mycelial morphology for intracellular intrusion. Furthermore, a shift from mononuclear spore to multinuclear spore phenotype can help evade capture by macrophages.

High-throughput NGS has significantly advanced the study of mucormycosis, providing insights into genome structure, drug resistance, and host-pathogen interactions.

## Contemporary case studies using high-throughput NGS

Here, we describe some case studies reporting the diagnosis of mucormycosis through NGS approaches. Shen et al. (2023) conducted a case study on a pediatric neuroblastoma patient with a complaint of rhino cerebral mucormycosis caused by *Lichtheimia ramosa*. Due to misleading diagnosis for detecting mucormycosis, there was a delay in administering Amphotericin B, the firstline antifungal agent used in clinical settings. The mNGS sequencing identified the pan-pathogenic fungal strain in the peripheral blood sample collected from the patient; this was followed by pertinent treatment.

Zhang et al. (2022) reported another case study of a patient with mucormycosis invasion of the central nervous system. Fungal mycelia and acid-fast bacteria were not detected in fluorescent staining and acid-fast staining, respectively. However, by conducting metagenomic sequencing with NGS approaches and enumeration of specific sequence numbers, the investigators successfully detected the presence of *Rhizomucor pusillus* and other species of *Rhizomucor*.

Wen et al. (2021) isolated metagenomic DNA from samples (peripheral blood, cerebrospinal fluid, and bronchoalveolar lavage fluid) derived from patients with lymphoblastic leukemia. The sequencing technology identified *Rhizomucor miehei* as the disseminating *Mucormycetes*. Accordingly, parenteral preparations of liposomes carrying Amphotericin B were administered. Subsequently, the general condition of the patient recovered along with improvement of left limb function.

The development of novel omics technologies has also discovered a long list of potential genes that were not previously associated with virulence, thus offering encouraging targets for designing novel therapeutics for mucormycosis. Lastly, the availability of various cellular and molecular techniques has facilitated the study of the genetic response elicited during host-pathogen interactions, highlighting the critical function of several regulatory genes.

## Conclusions

This article synthesizes recent developments in the genetic study of mucormycosis, focusing on omics-based methods. Previous studies have often relied on known virulence factors such as iron uptake mechanisms and cotH proteins. WGS has revealed numerous genes of unknown function. These genes, particularly those unique to *Mucormycetes*, merit further studies.

## Future possibilities

Omics research has revealed many unsolved questions and provided promising research directions. A key question is which fungal genes are activated during the pathogenetic process of mucormycosis. Because the infected samples predominantly contain host RNA, selective enrichment techniques are necessary. Murine models and RNA expression analysis can help reveal appropriate therapeutic targets and biomarkers.

The evolution of omics encompasses integration across genomics, transcriptomics, proteomics, and emerging fields such as immunomics and microbiomics (Dai and Shen 2022). Integrating knowledge-based and technology-based omics can advance mucormycosis research. Immunomics could enhance diagnosis, treatment, and vaccine development for mucormycosis.

Bioinformatics plays a key role in omics, offering tools for quality control, anomaly detection, interaction prediction, and network analysis. Advancing research on mucormycosis requires both wet lab innovations and dry lab computational techniques.

Integration of genomics, transcriptomics, and proteomics data, particularly immunological annotations, enables robust biomarker identification. Interactomics tools and programming languages such as Python can help elucidate protein–nucleic acid interactions (P–N interactions), supporting next-generation strategies for managing mucormycosis. The P–N interactions can reveal the behavioral aspects of *Mucorales*, including invasion of host tissues and resistance to antifungal drugs. Python enables bioinformatics and computational biology tools to model and analyze these interactions. The specific tools are used for (a) sequence analysis and (b) libraries such as Biopython, which can read, write, and manipulate DNA/RNA/protein sequences. Additionally, motif discovery, multiple sequence alignment, and annotation of key binding domains can be performed. Structural predictions tools such as MDAnalysis, PyMOL, or integration with AlphaFold can help predict 3D structure of protein-nucleic acid complexes. Moreover, the visualization of these structures could enable researchers to understand binding sites and interaction dynamics, which are the key factors to identify potential drug targets.

Contemporarily, machine learning integration can have Python’s ML libraries (e.g., scikit-learn, TensorFlow, and Keras), which allow to train models that predict interaction patterns from large biological datasets. The AI-powered molecular docking simulations can infer the interactions of antifungal drugs with fungal proteins. Systems biology and the network modeling approach using Python can model gene regulatory networks affected during mucormycosis. Libraries such as NetworkX can visualize how fungal genes interact with host immune genes, which is particularly useful in studying post-COVID immune dysregulation.

The integration of computational methods with various programming languages forms a solid foundation to establish fungal genomics and simulation of host-pathogen interactions. The funannotate and ChroQueTas toolchains define Python as a general-purpose central node that facilitates genome annotation, resistance gene identification, and mutation prediction. Python is further used to model *in silico* cytokine storms and loss of immune cells by using ordinary differential equations, agent-based modeling, and mixed simulations (Palmer and Stajich 2020; Talaei et al. 2021; Kareva et al. 2022; Bédard et al. 2025). Besides Python, R environment is required to run statistical genomics and transcriptomic analyses, with DESeq2 and edgeR being commonly used to determine differential gene expression of fungi in response to antifungal treatment (Robinson et al. 2010; Love et al. 2014). C/C++ represents the computing platform of choice to achieve rapid sequence alignment and resistance gene discovery, represented by the tools Bowtie2 and HMMER (Eddy 2011; Langmead and Salzberg 2012). Java-based frameworks, including Cytoscape to process network biology and GATK to determine variants, broaden the analytical domain (Shannon et al. 2003; McKenna et al. 2010). Concurrently, MATLAB has been widely applied within systems biology to execute multi-scale ODE/PDE models of infection dynamics and immune signaling circuits (Aldridge et al. 2006).

The integration of these complementing programming languages – genomic mining and immune simulation with Python, transcriptomic analysis with R, performance-critical genome operations with C/C++, variant and network analysis with Java, and systems-level modelling of the immune system with MATLAB – allows us to develop an integrated pipeline that maps fungal genotypes to host immune responses. These cross-platform, multi-scale computational approaches not only enhance identification of resistance-conferring mutations but also facilitate predictive modeling of the outcome of immune perturbation on fungal invasion. Overall, this integration approach offers a translational system to achieve mechanistic understanding and therapeutically relevant intervention within the context of fungal pathogenesis.
